# Epigenetic Regulation in Reproductive Medicine and Gynecologic Cancers

**DOI:** 10.1155/2010/567260

**Published:** 2011-03-10

**Authors:** Shi-Wen Jiang, Brian Brost, Sean Dowdy, Xing Xie, Fan Jin

**Affiliations:** ^1^Mercer University, Macon, GA 31207-0001, USA; ^2^Division of Maternal-Fetal Medicine, Department of Obstetrics and Gynecology, Mayo Clinic, USA; ^3^Division of Gynecologic Oncology, Department of Obstetrics and Gynecology, Mayo Clinic, USA; ^4^Women's Hospital, Zhejiang University School of Medicine, Zhejiang 310006, China; ^5^Zhejiang University, Hangzhou 310058, China

Recent progress in the field of epigenetics has provided a new study angle for our research efforts on reproductive medicine and gynecologic malignancies. We have acquired valuable insight into the regulatory mechanism and biological effects of DNA methylation and histone modification, the two major epigenetic pathways. The newly acquired knowledge effectively complements that gained from the genetic standpoint and holds great potential for the prevention, diagnosis, risk assessment, and treatment of these diseases. Specifically, the DNA methylation and imprinting mechanisms are implicated in fertilization, early embryonic development, placental function, and pathogenesis of preeclampsia and intrauterine growth retardation. Aberrant DNA methylation and chromatin modification lead to gene-specific silencing of numerous tumor suppressor genes, DNA repair genes, and steroid hormone receptors. This special issue presents a collection of peer-reviewed papers focusing on these areas. While the issue is not intended as an exhaustive representation of all of the potential topics, they nevertheless provide insightful and multifaceted information that we consider a pleasure to share with the readers.

This special issue includes 9 articles: three of which are related to the IGF-II imprinting in placenta and the effects of reproduction procedures on imprinting; two describe epigenetic mechanisms and genetic test for infertility; another paper documents the effects of in vitro maturation on histone acetylation in oocytes and early cleavage embryos; two address DNA methylation changes in cancers; one paper discusses rational design of primer for methylation assays.

In the first paper entitled “*Effects of in vitro maturation on histone acetylation in metaphase II oocytes and early cleavage embryos,*” Wang et al. document a reduced expression of histone acetyltransferase GCN5 (GCN5) and histone deacetylase 1 (HDAC1) in two-cell embryos but a normal level of these enzymes after the two-cell stage. The results indicate that *in vitro* maturation could affect protein and gene expression related to histone acetylation in oocytes and early cleavage embryos. However, by function of selection, parts of the changes could be recovered in late embryo development. 

In the second paper entitled “*Imprinting and promoter usage of insulin-like growth factor II in twin discordant placenta,*” Luo et al. analyze the imprinting and promoter usage of IGF-II in placenta of normal twins and twins with weight or phenotype discordance and conclude that promoter 3 specific LOI of the IGF-II gene may be closely related to phenotype discordance, but not to weight discordance. 

In the third paper entitled “*Oxidative stress and DNA methylation in prostate cancer,*” Donkena et al. present a comprehensive review on the effects of oxidative stress on DNA methylation and cancer progression, life style and diet as factors involved in ontogenesis and epigenetic interference for cancer prevention, and DNA methylation as a biomarker for cancer detection. Updates on the application of DNMT inhibitors to chemotherapy are also provided. 

In the fourth paper entitled “*Preimplantation genetic screening: an effective testing for infertile and repeated miscarriage patients?,*” Wang et al. compare results from different laboratories on preimplantation screening of aneuploidy and assess the efficacy, risks, and benefits of the procedure. They conclude that the use of preimplantation genetic screening should be reconsidered.

In the fifth paper entitled “*Study on the imprinting status of insulin-like growth factor II (IGF-II) gene in villus during 6–10 gestational weeks,*” Chen et al. compared the rate of loss of GF-II imprinting in the placental villous tissues between normal and abnormal embryo development and observed a significantly increased loss of imprinting in the abnormal group, suggesting that the imprinting status of IGF-II may be functionally related to embryo development.

In the sixth paper entitled “*Effects of assisted reproduction technology on placental imprinted gene expression,*” Katagiri et al. investigate the impact of assisted reproduction techniques (ART) on imprinted gene expression in human placenta. Different changes in the mRNA levels of imprinted genes are observed in the ART group compared with the spontaneous conception group, suggesting that ART may modify epigenetic status.

In the seventh paper entitled “*Specificity of methylation assays in cancer research: a guideline for designing primers and probes,*” Barekati et al. discuss the critical parameters to be considered for a rational design of PCR primers used for the detection of methylated DNA. The authors also provided applicable tools/algorithms and useful websites.

In the eighth paper entitled “*Epigenetic regulatory mechanisms associated with infertility,*” Minocherhomji et al. review the epigenetic mechanisms involved in spermatogenesis and infertility. Topics discussed in detail include the regulation and potential role of epigenetics in infertility by high-order chromatin organization, epigenetic control of genes associated with pericentromeric regions of chromosome 9 and Y, and noncoding RNAs. 

In the ninth paper entitled “Hypermethylation of SOX2 promoter in endometrial carcinogenesis”, Wong et al. report their studies on the methylation profiles of SOX2, a gene encoding the stem cell-related transcription factor SOX2 in endometrial carcinomas. Compared to normal control tissues, cancer tissues show hypermethylation and decreased expression of SOX2. The authors conclude that epigenetic silencing mechanisms may play a crucial role in transcriptional regulation of SOX2 and loss of SOX2 expression.

*Shi-Wen Jiang* *Shi-Wen Jiang* *Brian Brost* *Brian Brost* *Sean Dowdy* *Sean Dowdy* *Xing Xie* *Xing Xie* *Fan Jin* *Fan Jin*

